# Dangers of National Associations

**Published:** 1896-10

**Authors:** 


Dangers of National Associations. The National Veterinary Association of Great Britain is in anything but a flourishing condition, and much adverse criticism of its lack of growth, its absence of representative character, and the mutual admiration among its membership is indulged in. This should not be, and it can be readily remedied if those who criticise and feel that all this is true will only enter its ranks and make themselves a factor and a force in changing these conditions. We had a similar state of affairs in the U. S. V. M. A. once. But that day is gone never to return. Its programme is made up annually of new men; its conventions change largely each year; and in the severe financial times of 1896 it had the largest and most representative convention of veterinarians ever held in America, many of those present having travelled from 2000 to 4000 miles to attend its sessions, and all returned to their homes deeply impressed with its great usefulness and with an exalted opinion of the great worth of membership in its ranks. It is rapidly gathering within its fold every strong man of the profession and enlisting the efforts of every progressive veterinarian in America. And none look with more delight at these radical changes of progress than those who for many years sustained our organization when it was wholly local in character and national only in name. Take courage, brother veterinarians across the sea, and rise up in your strength and might. It will do you good; it will be a boon to your profession, and your association will advance and correct the evils of which you now complain.

